# Association of vitamin D-binding protein and vitamin D_3_ with insulin and homeostatic model assessment (HOMA-IR) in overweight and obese females

**DOI:** 10.1186/s13104-021-05608-6

**Published:** 2021-05-19

**Authors:** Leila Setayesh, Krista Casazza, Nariman Moradi, Sanaz Mehranfar, Habib Yarizadeh, Abbas Amini, Mir Saeed Yekaninejad, Khadijeh Mirzaei

**Affiliations:** 1 Department of Community Nutrition, School of Nutritional Sciences and Dietetics, Tehran University of Medical Sciences, P.O. Box: 14155-6117, Tehran, Iran; 2 Students Scientific Research Center (SSRC), Tehran University of Medical Sciences, Tehran, Iran; 3 Marieb College of Health and Human Services, Florida Gulf Coast University, Florida, FL USA; 4 Cellular and Molecular Research Center, Research Institute for Health Development, Kurdistan University of Medical Sciences, Sanandaj, Iran; 5 Department of Mechanical Engineering, Australian College of Kuwait, 13015 Safat, Kuwait; 6 Department of Epidemiology and Biostatistics, School of Public Health, Tehran University of Medical Sciences, Tehran, Iran

**Keywords:** Vitamin D binding protein, Vitamin D3, Obesity, Diabetes mellitus, Insulin, Homeostatic model assessment (HOMA-IR)

## Abstract

**Objective:**

Equivocal association the contribution of 25-hydroxyvitamin D (25(OH)D) and the well-accepted role of vitamin D-binding protein (VDBP) on bioavailability of 25(OH)D or its independent roles, has led to possible association of the VDBP in glucose metabolism. This study was conducted to evaluate the relationships among 25(OH)D, VDBP, glucose/insulin metabolism and homeostatic model assessment (HOMA-IR). Blood samples were collected from 236 obese and overweight women. VDBP and 25(OH)D levels, and biochemical parameters were measured using an enzyme-linked immunosorbent assay (ELISA). An impedance fat analyzer was utilized to acquire the body composition.

**Results:**

Using the multivariate linear regression, a reverse relationship was observed between VDBP and (HOMA-IR), such that women with higher VDBP displayed lower insulin resistance. The relationship was independent of age, body mass index, standardized energy intake and physical activity (p = 0.00). No significant relationship between 25(OH)D levels, FBS, body composition or insulin resistance were observed (p > 0.2). Current study observed that higher level of VDBP may be associated with lower levels of insulin and HOMA-IR, thus the evaluation of VDBP in diverse population groups seems to have significant clinical value in evaluating the prevalence of DM or early stage of glucose intolerance.

**Supplementary Information:**

The online version contains supplementary material available at 10.1186/s13104-021-05608-6.

## Introduction

The incidence and prevalence of prediabetes and type 2 diabetes mellitus (DM) represent a global public health concern. Over 500 billion individuals with DM were recognized in 2017 and more than 20 million new cases are annually diagnosed [1]. A more comprehensive understanding of interacting factors involved in impaired glucose control can assist prevention/intervention efforts. As the prevalence of DM continues to rise globally, the contemporary lifestyle factors (e.g., obesity and inactive lifestyles) accelerate its progression [2]. As investigations continue to uncover potential additional contributing factors, vitamin D bioavailability, which also displays racial/ethnic differences, has distributed significant interests among researchers.

In the circulation, the majority of 25(OH)D and following activation in the kidney 1,25(OH)2D are bound to vitamin D-binding protein (VDBP). About 1–2% is bound to albumin and a small fraction circulates in free unbound form [3–5]. VDBP integral in maintaining total and free 25(OH)D concentrations, [6–8] to some extent constrain some actions of vitamin D, through rendering it unavailable to act on its target cells [9, 10]. The identification of a receptor for 1,25‑dihydroxy vitamin D3 (25(OH)D) in the pancreatic β cells supports a role for vitamin D in insulin secretion and sensitivity [11]. It has been hypothesized that increased adipocyte size can lead to a reduction in vitamin D bioavailability due to higher sequestration and lower mobilization of vitamin D by large adipocytes [12]. Lower 25(OH)D in obese subjects has also been suggested due to altered hepatic synthesis and negative feedback mechanisms [13].

Notwithstanding, the relationship between vitamin D deficiency and glucose homeostasis necessitates the consideration of VDBP specially because of dramatic increase of diabetes [14] and vitamin D deficiency [15] in the Middle East countries. So, this is first cross-sectional study identifying and evaluating the relationship of 25(OH)D concentrations to the level of VDBP, and both to insulin resistance, HOMA, and FBS among overweight and obese women.

## Main text

### Methods

#### Population study

This cross-sectional study enrolled 236 healthy overweight and obese women (17–56 years of age) randomly selected from 25 health centers in Tehran. Women with any acute or chronic inflammatory disease as well as regular use of medications or nutritional supplements, including vitamins such as D, and minerals such as calcium, within two months preceding the enrollment of this study excluded. In the present study, we excluded the pregnant or lactating women and those with body weight fluctuations or specific nutritional regimens over the last year.

Bioelectric impedance assay (InBody 720, Korea) was utilized to calculate the body fat mass (BFM), fat mass (FM), body fat percentage (%), waist to hip ratio (WHR), and visceral fat area (VFA), applying a standardized procedure. All women arrived to the health centers with 8 h fasting, and blood collected and measurements were obtained between 8:00 a.m.–12:00 p.m. by an experienced nutritionist and same protocol. All anthropometric parameters were measured from participants which wore light clothes without shoes. A calibrated balance scale (Seca 711; Seca, Hamburg, Germany) was applied to characterize the body weight (kg) that was rounded to the nearest 0.1 kg. A wall-mounted stadiometer (Seca 711; Seca, Hamburg, Germany) was used for height (cm) (rounded to the nearest 0.5 cm). BMI (kg/m^2^) was calculated via body weight divided by squared height; BMI was in the range of 25–49 kg/m^2^, indicating on average the women were overweight or obese. Waist circumference (WC) was measured in cm, with a non-stretchable measuring tape between the lower edge of rib cage and the iliac crest, and rounded to the closest 0.1 cm.

All biochemical analyses on serum samples were performed with Glucose Oxidase Phenol 4-Aminoantipyrine Peroxidase (GOD/PAP) method and enzyme-linked immunosorbent assay (ELISA) method (Human insulin ELISA kit, DRG Pharmaceuticals, GmbH, Germany), respectively, in our laboratory at the University of Tehran. Data on insulin minimum detectable concentration was 1.76 mIU/mL, intra CV was 2.19%, and inter CV was 4.4%.

Vitamin D deficiency and insufficiency was defined as a serum concentration of vitamin D < 30 ng/mL, and normal level ≥ 30 ng/mL (≥ 75 nmoL/L). VDBP (R&D kit) and 25(OH)D (Bolden, United Kingdom) levels were measured using an enzyme-linked immunosorbent assay (ELISA).

Homeostatic model assessment (HOMA-IR) and HOMA-BS are the methods to count insulin resistance and beta-cell function.

Those indexes were computed by the following formula: HOMA-IR = [fasting plasma glucose (mmol/L) × fasting plasma insulin (mIU/L)]/22.5 and HOMA-BS = 20 × fasting insulin (μIU/ml)/fasting glucose (mmol/ml) − 3.5 [10].

#### Statistical analysis

Descriptive statistics of study covariates and outcomes were performed. Kolmogorov–Smirnov test was used for evaluating the data distribution. The visually inspection was used for normality of relevant variables levels, and the extreme data outliers were excluded. Results were presented in mean ± standard deviation (SD). The differences among the vitamin D and VDBP categories and outcomes were analyzed by independent t-test. A multivariate linear regression model was used to assess the association of HOMA, insulin concentration and FBS (coded as 0 for normal and 1 for disorder), along with VDBP level and 25(OH)D (response variable). A multivariate model was used to adjust the results with potential cofounders including age, plus BMI, total energy intake, and physical activity and reported with beta (β) and standard error (SE). p-values < 0.05 were considered as statistically significant. All statistical analyses were performed using SPSS® 21 (SPSS Inc., Chicago, USA).

### Results

The mean (SD) for age, BMI, height, body weight, vitamin D level, and VDBP of subjects were 36.49 (8.38) years, 31.04 (4.31) kg/m^2^, 161.38 (5.90) cm, 80.89 (12.45) kg, 67.184 (3.08) ng/mL, and 435.736 (77.507) nmol/mL, respectively (Additional file 1: Table S1). The anthropometrics, HOMA, insulin, and FBS of the population were reported under different groups based on 25(OH)D sufficiency, < or ≥ 30 ng/ml (Table 1). A statistically significant difference between vitamin D categories for insulin was observed (p = 0.03), as well as a marginal significant difference between vitamin D groups for HOMA (p = 0.05). Also, individuals were grouped based on 25(OH)D sufficiency, < or ≥ 20 ng/Ml, and results showed that individuals with higher levels of 25(OH)D (≥ 20 nmol/L) had lower body fat mass (p = 0.03) (Additional file 2: Table S2).

**Table 1:** Anthropometric measurements, FBS, HOMA, and insulin of participants grouped on the basis of vitamin D (ng/mL) and vitamin D binding groups (nmol/mL)

	Vitamin D status*			VDBP*		
	Serum level < 30 N = 74	Serum level ≥ 30 N = 162	p value**	Serum level ≤ 439.28 N = 118	Serum level > 439.28 N = 118	p value**
Age (years)	35.6 (8.17)	36.6 (8.49)	0.50	35.8 (8.01)	36.6 (9.02)	0.11
Weight (kg)	81.5 (11.9)	81.0 (12.6)	0.38	79.5 (10.9)	82.4 (13.4)	0.09
Height (cm)	162.1(5.52)	161.5 (5.52)	0.43	161.4 (5.85)	161.9 (5.52)	0.38
BMI (kg/m^2^)	31.1 (4.14)	31.0 (4.5)	0.36	30.5 (3.7)	31.5 (4.83)	**0.04**
Body fat mass (%)	34.6 (8.10)	34.0 (9.04)	0.11	33.1 (7.69)	35.1 (9.46)	0.14
Percent body fat (%)	47.1 (5.69)	46.8 (5.48)	0.30	41.0 (5.80)	42.0 (5.26)	0.49
WHR (cm)	0.94 (0.05)	1.50 (7.19)	0.26	1.64 (8.04)	0.93 (0.05)	0.05
WC (cm)	100.3 (9.93)	98.9 (9.94)	0.19	98.0 (9.04)	100.2 (10.7)	0.10
FBS (mg/dL)	85.9 (8.55)	88.1(9.75)	0.69	87.1(10.2)	88.1 (9.31)	0.97
HOMA-BS (mg/dL)	0.00(1.29)	0.20(0.99)	0.06	0.17(1.25)	0.06(1.13)	0.15
HOMA-IR (mg/dL)	3.39 (1.02)	3.40 (1.82)	0.05	3.56 (1.94)	3.25 (1.14)	**0.03**
Insulin (mIU/L)	15.6 (4.32)	15.6 (7.23)	**0.03**	16.23 (7.512)	14.9 (14.9)	**0.03**
VDBP (μg/mL)	441.5 (72.2)	436.8 (80.6)	0.75	–	–	–
Vitamin D (ng/mL)	–	–	–	70.1 (47.3)	64.6 (47.4)	0.72

The population was presented under different groups on the basis of 25(OH)D, < or ≥ 30 ng/mL. The population was presented under different groups on the basis of VDBP median Serum level > 439.28 or Serum level ≤ 439.28. (nmol/mL)

*BMI* body mass index, *WHR* waist-hip ratio, *WC* waist circumference, *FBS* fasting blood sugar, *VDBP* vitamin D-binding protein, *SD* standard deviation, *HOMA* homeostatic model Assessment-Insulin resistance

^*^Results were expressed with mean ± standard deviation (SD)

^**^p values in bold denote significant differences (p < 0.05)

Median VDBP (≤ 439.28 nmol/L and > 439.28 nmol/mL) was used for further group-wise comparisons (Table 1). HOMA and insulin were positively related with across the categories of VDBP (p < 0.05); wherein individuals with higher levels of VDBP (> 439.28 nmol/L) had higher HOMA and insulin. Additionally, a marginally significant association was observed between the two groups of VDBP for BMI (p = 0.04) and WHR (p = 0.05).

The correlation between the level of 25(OH)D and age, anthropometrics, HOMA, insulin, and glucose are shown in Table 2. No significant association was observed between 25(OH)D level and the outcome variables from linear regression analysis in both crude and adjusted models (p > 0.1). The relationship among VDBP age, anthropometrics, HOMA, insulin, and glucose using linear regression analysis is presented in Table 3. VDBP was inversely related to HOMA and insulin concentration in serum in the crude model (p < 0.05), wherein individuals with greater VDBP had lower HOMA and insulin levels. The correlation of VDBP and insulin level (β = − 0.04, p = 0.01), HOMA (β = − 0.00, p = 0.01), was not affected by potential cofounders of age (years), BMI (kg/m^2^), standardized energy intake (kcal), and physical activity.

**Table 2:** Association of vitamin D levels (ng/mL) with the studied variables

	*Model 1			**Model 2		
	Β	SE	p	Β	SE	p
Age (years)	0.00	0.01	0.46	0.01	0.01	0.26
Height (cm)	0.00	0.00	0.27	0.01	0.00	0.27
BMI (kg/m^2^)	− 0.00	0.00	0.64	− 0.00	0.00	0.49
WHR (cm)	0.00	0.00	0.53	0.00	0.00	0.47
WC (cm)	− 0.00	0.01	0.58	− 0.00	0.00	0.85
FBS (mg/dL)	0.02	0.01	0.09	0.01	0.01	0.18
Insulin (uIU/mL)	0.01	0.01	0.29	0.01	0.01	0.36
HOMA-BS(mg/dL)	− 4.99	0.00	0.98	− 0.00	0.00	0.76
HOMA-IR (mg/dL)	0.00	0.00	0.28	0.00	0.00	0.27
VDBP (μg/mL)	− 0.04	0.10	0.70	− 0.11	0.12	0.35

*BMI* body mass index, *WHR* waist-hip ratio, *WC* waist circumference, *FBS* fasting blood sugar, *VDBP* vitamin D-binding protein, *SD* standard deviation, *HOMA* homeostatic model Assessment-Insulin resistance

Linear regression was applied to assess the correlation

^*^Model 1: Crude Model

^**^Model 2: Adjusted for age (year), body mass index (kg/m^2^), standardized energy intake (kcal), physical activity

**Table 3:** Association of VDBP levels with FBS, HOMA and Insulin level (μg/mL)

	*Model 1			**Model 2		
	Β	SE	p	β	SE	p
Age (year)	0.00	0.00	0.44	0.01	0.00	0.14
Height (cm)	0.00	0.00	0.98	0.00	0.00	0.67
BMI (kg/m^2^)	0.00	0.00	0.86	− 0.00	0.00	0.76
WHR (CM)	0.00	0.00	0.93	− 0.00	0.00	0.88
WC (cm)	− 0.00	0.00	0.84	− 0.00	0.00	0.44
FBS (mg/dL)	− 0.00	0.00	0.92	− 0.00	0.00	0.56
Insulin (mIU/L)	− 0.01	0.00	**0.03**	− 0.01	0.00	**0.01**
HOMA-BS(mg/dL)	− 0.00	0.00	0.60	− 0.05	0.00	0.95
HOMA-IR (mg/dL)	− 0.00	0.00	**0.02**	− 0.00	0.00	**0.01**
Vitamin D (ng/mL)	− 0.01	0.04	0.70	− 0.04	0.04	0.35

A bold p values donate a significant difference (p < 0.05)

*BMI* body mass index, *WHR* waist-hip ratio, *WC* waist circumference, *FBS* fasting blood sugar, *VDBP* vitamin D-binding protein, *SD* standard deviation, *HOMA* homeostatic model Assessment-Insulin resistance

Linear regression was applied to assess the correlation

^*^Model 1: Crude Model

^**^Model 2: Adjusted for age (year), body mass index (kg/m^2^) and standardized energy intake (kcal), physical activity

### Discussion

In this study on overweight and obese women in the Middle East (n = 236), VDBP was inversely correlated with insulin dynamics, such that higher VDBP was observed in conjunction with lower concentration of insulin and HOMA-IR. About 68% of the women had vitamin D sufficiency as defined by 25(OH)D > 30, and there was no association between 25(OH)D and studied variables observed. Furthermore, it was no association between age and anthropometric indicators including BMI, height, WHR, and WC with vitamin D and VDBP categories.

To date, the contribution of VDBP to insulin dynamics are inconsistent. Negar Naderpoor et al. conducted a research on PCOS women to assess the relationship between VDBP and insulin resistance in Australia; they reported no significant association [16]. On the other hand, Pelczyńska et al. reported that free and bioavailable fraction correlated with particular components of the syndrome, and VDBP concentration, also negatively and independently related with some parameters in those with metabolic syndrome [17]. Another evaluation of the relationship identified an association between VDBP and insulin resistance which appeared to be moderated by race [18]. Similar to the results from Ashraf et al. suggested that VDBP concentrations are inversely associated with insulin resistance and insulin level. They also showed that that VDBP levels may change to maintain adequate concentrations bioavailable 25(OH)D [18]. Collectively, these studies along with our findings in an underreported population suggest a potential clinical relevance for VDBP. Plausibly, VDBP may have important role in vitamin D bioavailability and conceivably underpinning insulin dynamics.

VDBP is a well-recognized and major carrier for both 25(OH)D and 1,25 (OH) D [19] in the circulation, which is produced and discharged by hepatic cells [20]. The amount of VDBP can affect the balance between free and bonded fractions and direct the transport of hormone to the target tissues. The higher level of VDBP can decrease the free and bioavailable 25(OH)D concentration for target cell and eventually induce the symptom of vitamin D deficiency in body [15, 21, 22]. Fat distribution (i.e., visceral adiposity) is an adverse contributor to glucose metabolism. Hypothetically, visceral adiposity interrupts vitamin D transport in blood and its metabolism; further, via multiple feedback pathways, the DM risk can be increased [23]. In addition, the development of DM may be prognosticated via a decreased 25(OH)D concentration in serum [24–26]. However, the large randomized clinical trials could not confirm the beneficial effect of vitamin D supplementation on the improvement of glucose intolerance and DM [27]. These equivocal outcomes may be due to different levels of VDBP concentration in serum which was shown in current study, regardless of 25(OH)D, VDBP was inversely related with HOMA and insulin levels that are the predictor of developing and inducing DM.

Research results reported that the level of VDBP could be increased in the response to inflammation definitely to the interleukin-6, therefore, due to the more prevalent of systematic inflammation in the obese and overweight individuals, the level of this carrier would be augmented [28]. Further analyses including inflammatory markers are warranted [29].

We observed that the insulin levels and VDBP was inversely related even after the correction for BMI and body fat. It was proposed that the synthesis of VDBP would be suppressed by insulin [18, 30]. On the other hand, based on previous data, the decreased level of 25(OH)D had no impact on the reduction of bioavailable and free concentration in the Australian population. Additionally, the negative relationship of total 25(OH)D with insulin resistance factors was clarified [18]. With the presence of VDBP and vitamin D receptor in animal pancreatic tissue, it is rationalized that the 25(OH)D concentration in beta cells can be controlled by VDBP with the ultimate major impact on insulin discharge [18]. Furthermore, it is thought that VDBP, by regulating the amount of active vitamin D in β-cells of the pancreas, may affect insulin secretion, and thus affect the prevalence of insulin resistance and type-2 diabetes mellitus [18]. In spite of the requirement to further examinations, it has been assumed that inherited gene polymorphisms, such as VDBP, 1α-hydroxylase genes, and vitamin D receptor, have important correlations with vitamin D status in balancing and mediating the glucose homeostasis and insulin sensitivity [31–34].

### Conclusion

We showed an independent association between VDBP and insulin and HMOA. The study findings suggest a potential role of VDBP as a putative predictor of insulin resistance among overweight and obese women. Further prospective trials are warranted to evaluate the effect of higher VDBP levels as a root cause of the dysregulation of glycemic profile.

## Limitation

The current study has several limitations. According to the observational nature of the study, the prediction of exact cause-effect influence of VDBP and vitamin D concentrations on glycemic profile for other ethnicities was not practiced. The low number of enlisted participants and not including male for evaluation (possible impact of estrogen on VDBP) were considered as other drawbacks of this study. Lastly, we could not compute free and bioavailable vitamin D, due to the absence of blood albumin data.

## Supplementary Information

**Additional file 1: Table S1.** Anthropometric parameters among target population.

**Additional file 2: Table S1.** Anthropometric and FBS, HOMA, and Insulin level of participants grouped on the basis of vitamin D (ng/mL).

## Data Availability

The data that support the findings of this study are available from the corresponding author, upon reasonable request.

## Abbreviations

BMI: Body mass index
WHR: Waist-hip ratio
FMI: Fat mass index
FFMI: Fat-free mass index
WC: Waist circumference
HOMA-IR: Homeostatic model assessment
FBS: Fasting blood sugar
VDBP: Vitamin D-binding protein
