# Supplementary material for: Association of vitamin D-binding protein and vitamin D3 with insulin and homeostatic model assessment (HOMA-IR) in overweight and obese females
Source: BMC Res Notes. 2021 May 19;14:193. doi: 10.1186/s13104-021-05608-6 (PMC8136187; doi:10.1186/s13104-021-05608-6)
Supplement: Supplementary file 1 — Additional file 1: Table S1. Anthropometric parameters among target population. [file 13104_2021_5608_MOESM1_ESM.docx]

**Table S1.** Anthropometric parameters among target population

| Parameters | | Minimum | | Maximum | | Mean | | SD | |
| --- | --- | --- | --- | --- | --- | --- | --- | --- | --- |
| Age (year) | | 17.00 | | 56.00 | | 36.49 | | 8.38 | |
| Weight (kg) | | 59.50 | | 136.60 | | 80.89 | | 12.45 | |
| Height (cm) | | 142.00 | | 179.00 | | 161.38 | | 5.90 | |
| BMI (kg/m^2^) | | 24.20 | | 49.60 | | 31.04 | | 4.31 | |
| Percent body fat (%) | | 15.00 | | 54.30 | | 41.53 | | 5.48 | |
| WHR (cm) | | 0.81 | | 1.08 | | 0.93 | | 0.05 | |
| Body fat mass (%) | | 19.40 | | 74.20 | | 34.04 | | 8.69 | |
| Obesity degree (%) | | 29.40 | | 231.00 | | 143.69 | | 21.51 | |
| WC (cm) | | 80.10 | | 136.00 | | 99.01 | | 10.05 | |
| Energy (kcal) | | 1028.98 | | 4192.72 | | 2613.04 | | 44.07 | |
| VDBP (μg/mL) | | 185.34 | | 798.39 | | 435.74 | | 77.51 | |
| FBS (mg/dL) | | 67 | | 137 | | 87.49 | | 9.64 | |
| Insulin (mIU/L) | | 6.67 | | 65.89 | | 15.59 | | 6.02 | |
| HOMA-BS(mg/dL) | | -2.42 | | 4.71 | | 0.06 | | 1.67 | |
| HOMA-IR (mg/dL) | | 1.26 | | 16.59 | | 3.40 | | 1.52 | |
| Vitamin D (ng/mL) | | 2.90 | | 191.40 | | 67.18 | | 3.08 | |

BMI, body mass index; WHR, waist-hip ratio; WC, waist circumference; FBS, fasting blood sugar; VDBP, vitamin D-binding protein; SD, standard deviation; HOMA, homeostatic model Assessment-Insulin resistance.
