# Supplementary material for: Association of vitamin D-binding protein and vitamin D3 with insulin and homeostatic model assessment (HOMA-IR) in overweight and obese females
Source: BMC Res Notes. 2021 May 19;14:193. doi: 10.1186/s13104-021-05608-6 (PMC8136187; doi:10.1186/s13104-021-05608-6)
Supplement: Supplementary file 2 — Additional file 2: Table S1. Anthropometric and FBS, HOMA, and Insulin level of participants grouped on the basis of vitamin D (ng/mL). [file 13104_2021_5608_MOESM2_ESM.docx]

**Table S2.** Anthropometric and FBS, HOMA, and Insulin level of participants grouped on the basis of vitamin D (ng/mL)

| Variable | Serum level <20  N=74 | Serum level≥ 20  N=162 | P value |
| --- | --- | --- | --- |
| Age (years) | 34.74(9.04) | 36.54(8.28) | 0.26 |
| Weight (kg) | 81.99 (9.73) | 81.03(12.72) | 0.06 |
| Height (cm) | 162.46(6.56) | 161.57(5.57) | 0.41 |
| BMI(kg/m2) | 31.08 (3.73) | 31.05(4.48) | 0.31 |
| body.fat.mass(%) | 34.50 (6.70) | 34.15 (9.04) | **0.03** |
| percent.body.fat(%) | 42.41 (4.18) | 41.39 (5.80) | 0.07 |
| WHR(cm) | 0.94(0.05) | 1.38(6.42) | 0.44 |
| WC(cm) | 100.31 (8.47) | 99.14 (10.16) | 0.08 |
| FBS(mg/dL) | 86.34(6.84) | 87.57(9.79) | 0.26 |
| HOMA-BS(mg/dL) | 0.26(0.99) | 0.03(1.23) | 0.17 |
| HOMA-IR(mg/dL) | 3.41 (1.11) | 3.39 (1.68) | 0.41 |
| Insulin (mIU/l) | 15.88(4.78) | 15.10(5.03) | 0.71 |
| VDBP(μg/ml) | 436.78(64.04) | 437.96(74.23) | 0.93 |

The population was presented under different groups on the basis of 25(OH)D, < or ≥20 ng/Ml.

BMI, body mass index; WHR, waist-hip ratio; WC, waist circumference; FBS, fasting blood sugar; VDBP, vitamin D-binding protein; SD, standard deviation; HOMA, homeostatic model Assessment-Insulin resistance

Results were expressed with mean ± standard deviation (SD)

p values in bold denote significant differences (p<0.05).
